# Prevalence and correlates of suicide attempt among Chinese individuals receiving methadone maintenance treatment for heroin dependence

**DOI:** 10.1038/s41598-019-52440-x

**Published:** 2019-10-30

**Authors:** Bao-Liang Zhong, Wu-Xiang Xie, Jun-Hong Zhu, Jin Lu, Hongxian Chen

**Affiliations:** 1 Department of Psychiatry, The Second Xiangya Hospital, Central South University, Changsha, Hunan Province China; 2 China National Clinical Research Center on Mental Disorders (Xiangya), Changsha, Hunan Province China; 3 Affiliated Wuhan Mental Health Center, Tongji Medical College of Huazhong University of Science & Technology, Wuhan, Hubei Province China; 4 Peking University Clinical Research Institute, Peking University Health Science Center, Beijing, China; 5 Department of Psychiatry, The First Affiliated Hospital of Kunming Medical University, Kunming, Yunnan Province China

**Keywords:** Addiction, Epidemiology

## Abstract

To date, there have been very limited studies regarding the clinical epidemiology of attempted suicide in Chinese individuals with heroin-dependence. The objective of this study was to examine the prevalence and correlates of suicide attempt in Chinese individuals receiving methadone maintenance treatment for heroin dependence. Demographic, clinical, and psychosocial data of 603 methadone-maintained patients with heroin dependence were collected with a standardized self-administered questionnaire. The presence of suicide attempt and antisocial personality disorder was assessed by using a single question and the Mini-International Neuropsychiatric Interview 5.0. The one-month and lifetime prevalence rates of suicide attempt were 9.5% and 34.2%, respectively. In multivariable logistic regression, lifetime suicide attempt was significantly associated with female gender (OR = 2.81), being 20–39 years old (OR = 2.73), an education level of primary school or lower (OR = 2.07), poor economic status (OR = 3.06), injecting heroin before methadone maintenance treatment (OR = 2.92), depressive symptoms (OR = 3.46), anxiety symptoms (OR = 1.88), and antisocial personality disorder (OR = 2.85). Suicide attempt is very prevalent among Chinese individuals receiving methadone maintenance treatment for heroin dependence. Services for patients with heroin dependence in methadone maintenance treatment clinics in China should include psychosocial supports, periodic screening for suicide attempt and other suicidal behaviors and, when needed, psychiatric treatment and crisis intervention.

## Introduction

Persons who use heroin and other opioids are at greater risk for premature deaths, with suicide representing a substantial and important contributor to the overall excess mortality seen in opioid users^1–3^. For example, in Taiwan, the all-cause mortality and suicide death rates are over seven and 15 times higher in individuals who use heroin (IUH) than those in the general population, respectively^4,5^. It has been estimated that approximately 3–35% deaths among IUH could be ascribed to suicide^6^. Considering the “hidden” suicides among deaths of IUH certified as opioid overdose and accidents^4,7–9^, the percentage of deaths by suicide among IUH would be higher than the above estimates.

IUH are also at elevated risk for other suicidal behaviors, including ideation, plan, and attempt^10,11^. Among all known risk factors for suicide, including these non-fatal suicidal behaviors, a prior attempted suicide is one of the most robust predictor of completed suicide^12–15^. Evidence also shows that some suicide-preventive measures aimed at individuals who previously attempted suicide are effective to reduce suicides^16,17^. Therefore, a better understanding on the clinical epidemiology of suicide attempt may help the early identification of IUH at elevated risk for suicide and would facilitate the development of preventive measures for suicide in this high-risk population.

Given the large number of persons with opioid dependence and serious consequences associated with opioid dependence, opioid dependence remains the largest contributor to the current global burden of disease caused by illicit drug use^18,19^. In Western countries, the epidemiology of suicide attempt in individuals with opioid dependence has been extensively studied^10,20–25^. These studies reported a wide range of lifetime prevalence of attempted suicide in patients with opioid dependence (8.0–48.0%) and a variety of risk factors associated with suicide attempt, including female gender, unemployment, depression, post-traumatic stress disorder, personality disorder, a high degree of aggression/impulsivity, childhood trauma, alcoholism, and addiction severity.

In China, although the most prevalent type of drug used has turned from opioids to synthetic drugs in recent years, the number of individuals who use opioids is still quite large. For example, in 2016, there were a total of approximately one million IUH, accounting for 38.1% of the total population of illicit drug users in China^26^. To reduce harms caused by opioid use disorders, China initiated its methadone maintenance treatment (MMT) program in 2004, and, until now, it has had the world’s largest MMT network^27,28^. According to the official data, by 2016, a total of 162000 IUH were receiving treatment in 789 community-based MMT clinics distributed in all provinces of China^29^. Although MMT is effective in reducing withdrawal symptoms and social harms resulting from heroin use and improving quality of life of individuals with heroin dependence (IHD)^30^, empirical studies still reported high prevalence rates of psychosocial and physical problems in Chinese IHD receiving MMT such as depression, loneliness, and pain^27,31,32^.

In mainland China, studies on the epidemiology of attempted suicide in IHD are limited. To the best of our knowledge, only five studies, all published in Chinese language journals, have examined the prevalence of suicide attempt in Chinese IHD: two were conducted in compulsory detoxification centers and three conducted in MMT clinics^33–37^. Nevertheless, lifetime prevalence estimates reported by them varied considerably, ranging from 0.66% to 30.7%, a 47-fold difference. Moreover, because of the small numbers of suicide attempters identified in these studies (median: 17, range: 3–39), they only speculated possible reasons for attempted suicide such as stigma associated with being an “addict”, depressive emotion, poor relationship with family members, hopelessness, and inadequate social support. Therefore, findings on the epidemiology of suicide attempt in Chinese IHD, particularly correlates, are very preliminary.

Given the wide variations in the prevalence of suicide attempt and the lack of empirical evidence on the correlates of suicide attempt among IHD in China, this study was set out to determine the prevalence and correlates of suicide attempt among Chinese IHD in MMT settings.

## Materials and Methods

### Subjects

The current study was a secondary data analysis based on data from a cross-sectional study, which examined quality of life, mental health problems, non-suicidal self-injury, and non-fatal suicidal behaviors of Chinese patients receiving MMT in Wuhan, Hubei province, China, between June 2009 and July 2010^27,28^. Wuhan is the most populous metropolitan area in Central China with a population of almost 11 million persons. Three city-owned MMT clinics were chosen as our study sites because of their large numbers of outpatient visits in this city (approximately 100–300 visits per clinic per day). Patients who were 20 years and older, met DSM-IV diagnostic criteria for lifetime heroin dependence, were taking liquid methadone at these clinics at the time of the survey, and agreed to participate, were consecutively recruited. We excluded patients with current alcohol dependence, brain organic mental disorders, and psychotic symptoms, as well as those who were too physically ill to complete the questionnaire or interview. Data on DSM-IV diagnoses of current alcohol dependence and brain organic mental disorders, psychotic symptoms, and physical conditions were obtained by a careful manual review of patients’ paper medical records. During the review process, a checklist was used to collect these data and the above inclusion and exclusion rules were strictly applied (Supplementary Table S1).

### Assessments

A self-administered questionnaire specifically designed for this study was used to collect data on patients’ demographics, clinical characteristics, and psychosocial factors.

Demographic variables included age, gender, education, marital status, employment status, and self-rated economic status (good, fair, and poor).

Clinical data were main route of past heroin administration, length of past heroin use, duration of MMT, dose of methadone, and pain. The five-point Verbal Rating Scale was used to evaluate pain intensity. The scale only has a single question: “Overall, how intense is your pain now?”, which is answered on a five-point scale: 1 = none, 2 = mild, 3 = moderate, 4 = severe, 5 = very severe. This measure of pain is widely used in prior studies and subjects who rate their pain as “moderate”, “severe”, or “very severe” are categorized as having clinically significant pain^27,38,39^.

Psychosocial factors included depressive and anxiety symptoms and loneliness. Depressive and anxiety symptoms were assessed with the validated Chinese version of Zung’s Self-rating Depression Scale (SDS) and Zung’s Self-rating Anxiety Scale (SAS), respectively^40^. Both scales have the same number of items (n = 20), and the same total score, ranging between 0 and 80. We defined clinically significant depressive and anxiety symptoms according to the recommended cut-off values of SDS and SAS, which are ≥ 40 and ≥43 for the Chinese population, respectively^40^. Loneliness was measured with a single question (How often do you feel lonely?) on a 5-point Likert scale: 1 = always, 2 = often, 3 = sometimes, 4 = seldom, 5 = never. This question is a commonly used measure of loneliness, which is directly adapted from previous studies^41–43^. Consistent with prior studies^31,43,44^, the five-category loneliness variable was transformed into a dichotomous variable: “lonely” (“always”, “often”, and “sometimes”) and “not lonely” (“seldom” and “never”).

After the completion of the questionnaire, patients were interviewed with the antisocial personality disorder (APD) module of the Chinese Mini-International Neuropsychiatric Interview (MINI) 5.0 by trained psychiatrists^45^. Because there is evidence that the comorbid APD confers additional risk of suicide in persons with substance use disorders^46^, MINI 5.0 was used to ascertain the presence of lifetime APD in IHD. The Chinese MINI 5.0 has been shown to be valid for diagnosing APD in MMT patients^47^.

The outcome of this study, suicide attempt, was measured with the question adapted from the National Comorbidity Survey^48^: “Have you ever attempted suicide?”. If the patient endorsed it, he/she would be recorded as having lifetime suicide attempt. Patients with lifetime attempt would be further asked: “When was the last time?”. If a response was affirmative for any time during the past month, the patient would be recorded as having one-month suicide attempt.

The survey investigators were six treating psychiatrists of patients in the three MMT clinics. They were arranged to interview patients with MINI and read out questions for subjects who had difficulty in completing the questionnaire.

### Statistical analysis

Prevalence rates of lifetime and one-month suicide attempt were calculated. Demographic, clinical, psychosocial, and personality characteristics of IHD with and without suicide attempt were described and compared by Chi-square test. Multivariable logistic regression model with the “Enter” method was used to identify factors significantly associated with suicide attempt. Suicide attempt was entered as the dependent variable, and all demographic, clinical, psychosocial, and personality factors were entered simultaneously as independent variables. Odds ratios (ORs) and 95% confidence intervals (CIs) were used to quantify the associations between factors and suicide attempt. The statistical significance level was set at P ≤ 0.05 (two-sided). SPSS software version 15.0 package was used for analyses.

### Ethics approval

All study procedures were conducted in accordance with the ethical standards of the 1975 Helsinki Declaration. Written informed consent was obtained from each participant and declarations of anonymity and confidentiality had been made before the start of data collection. The institutional reviewed board of Wuhan Mental Health Center approved the study protocol.

## Results

**Figure 1:**
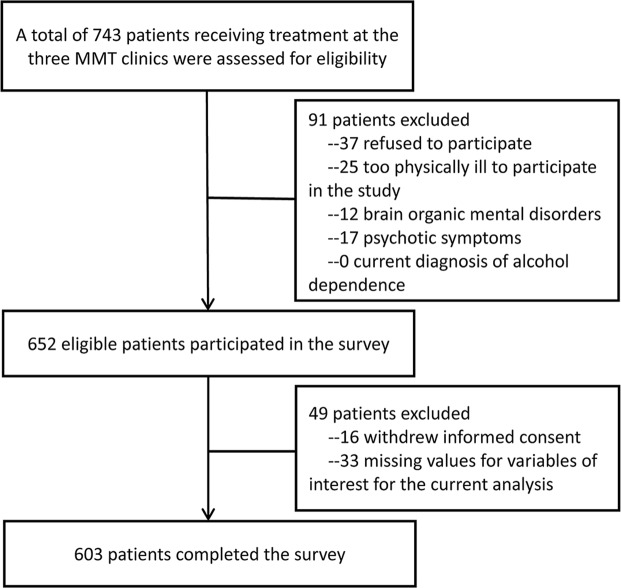
Flowchart of subject recruitment.

At the time of the survey, there were a total of 743 patients receiving MMT in the three clinics. All these patients were assessed for eligibility and 652 were eligible for this study. No patients were excluded due to a current diagnosis of alcohol dependence. A final sample of 603 patients (92.5%) successfully completed the survey. The subject recruitment process is shown in Fig. 1.

In this sample of IHD from MMT clinics, 69.8% were men and the mean age was 38.1 years (range: 21–59, standard deviation [SD] = 7.0). Before admission to MMT, the main route of heroin use was injection (84.1%) and the average duration of heroin use was 10.8 years (SD = 8.7). At the time of the survey, these patients had taken methadone for a mean duration of 24.6 months (SD = 11.0) at an average dosage of 69.5 mg per day (mg/d) (SD = 29.6). Table 1 shows detailed demographic, clinical, psychosocial, and personality characteristics of our sample of IHD receiving MMT.

**Table 1:** Characteristics of Chinese individuals receiving methadone maintenance treatment (MMT) for heroin dependence and prevalence rates of attempted suicide by different characteristics.

Characteristics		n	One-month attempted suicide				Lifetime attempted suicide			
			Number	%	χ2	P	Number	%	χ2	P
Gender	Male	412	35	8.5			127	30.8		
	Female	191	22	11.5	1.259	0.262	79	41.4	8.475	0.004
Age (years)**	20–39	327	41	12.5			131	40.1		
	40–59	276	16	5.8	7.495	0.006	75	27.2	10.737	0.001
Education	Primary school or lower	64	16	25.0			37	57.8		
	Middle school or higher	539	41	7.6	18.255	<0.001	169	31.4	18.875	<0.001
Marital status	Married	295	25	8.5			94	31.9		
	Non-married*	308	32	10.4	0.48	0.488	112	36.4	1.529	0.616
Employment status	Yes	317	20	6.3			100	31.5		
	No	286	37	12.9	6.701	0.010	106	37.1	1.898	0.168
Self-rated economic status	Good	149	7	4.7			40	26.8		
	Fair	314	27	8.6			99	31.5		
	Poor	140	23	16.4	11.722	0.003	67	47.9	13.495	0.001
Route of past heroin administration	Smoking	96	1	1.0			11	11.5		
	Injecting	507	56	11.0	9.16	0.002	196	38.7	21.701	<0.001
Duration of past heroin use (years)**	≤10	373	41	11.0			119	31.9		
	>10	230	16	7.0	2.535	0.111	87	37.8	2.049	0.152
Duration of MMT (months)**	≤24	240	20	8.3			71	29.6		
	>24	363	37	10.2	0.546	0.460	145	39.9	6.657	0.010
Methadone dose (md/d)**	≤70	273	30	10.9			110	40.3		
	>70	330	27	8.2	1.797	0.180	96	29.1	7.428	0.006
Depressive symptoms	No	399	21	5.3			128	32.1		
	Yes	204	36	17.6	22.586	<0.001	78	38.2	2.495	0.114
Anxiety symptom	No	384	20	5.2			105	27.3		
	Yes	219	37	16.9	18.285	<0.001	101	46.1	18.853	<0.001
Pain	No	280	23	8.2			85	30.4		
	Yes	323	34	10.5	0.749	0.387	121	37.5	3.286	0.070
Loneliness	No	226	11	4.9			79	35.0		
	Yes	337	46	13.6	14.109	<0.001	126	37.4	0.328	0.567
Antisocial personality disorder	No	381	17	4.5			97	25.5		
	Yes	222	40	18.0	29.212	<0.001	109	49.1	33.679	<0.001

*”Non-married” includes never-married, remarried, cohabitating, separated/divorced, and widowed.

**Continuous variables were dichotomized by a median split approach.

Altogether, 57 and 206 patients attempted suicide during the past month and at some time prior to the interview, respectively. The corresponding one-month and lifetime prevalence rates of suicide attempt were 9.5% (95%CI: 7.1–11.8%) and 34.2% (95%CI: 30.4–37.9%), respectively.

Results of comparisons between patients with and without suicide attempt (Table 1) show that, patients with one-month suicide attempt were significantly more likely to be 20–39 years old (P = 0.006), have an education level of primary school or lower (P < 0.001), be unemployed (P = 0.010), rate their economic status as “poor” (P = 0.003), inject heroin before MMT (P = 0.002), be depressed (P < 0.001), be anxious (P < 0.001), feel lonely (P < 0.001), and have APD (P < 0.001); while patients with lifetime suicide attempt were significantly more likely to be females (P = 0.004), be 20–39 years old (P = 0.001), have an education level of primary school or lower (P < 0.001), rate their economic status as “poor” (P = 0.001), inject heroin before MMT (P < 0.001), have a MMT duration of more than 24 months (P = 0.010), take a methadone dose of lower than 70 mg/d (P = 0.006), feel anxious (P < 0.001), and have APD (P < 0.001).

In multivariable logistic regression analyses (Table 2), one-month suicide attempt was significantly associated with female gender (OR = 3.37, 95%CI = 1.27–8.99, P = 0.015), being 20–39 years old (OR = 4.98, 95%CI = 1.95–12.66, P = 0.001), marital status of “non-married” (OR = 4.88, 95%CI = 1.93–12.35, P = 0.001), injecting heroin before MMT (OR = 4.60, 95%CI = 1.45–28.51, P = 0.036), a duration of heroin use of more than 10 years (OR = 3.24, 95%CI = 1.19–8.77, P = 0.021), anxiety symptoms (OR = 3.34, 95%CI = 1.12–9.98, P = 0.031), loneliness (OR = 5.02, 95%CI = 1.79–14.03, P = 0.002), and APD (OR = 5.86, 95%CI = 2.19–15.66, P < 0.001); while lifetime suicide attempt was significantly associated with female gender (OR = 2.81, 95%CI = 1.69–4.69, P < 0.001), being 20–39 years old (OR = 2.73, 95%CI = 1.66–4.48, P < 0.001), an education level of primary school or lower (OR = 2.07, 95%CI = 1.01–4.27, P = 0.048), poor economic status (OR = 3.06, 95%CI = 1.47–6.35, P = 0.003), injecting heroin before MMT (OR = 2.92, 95%CI = 1.38–6.13, P = 0.005), depressive symptoms (OR = 3.46, 95%CI = 1.70–7.04, P = 0.001), anxiety symptoms (OR = 1.88, 95%CI = 1.06–3.35, P = 0.032), and APD (OR = 2.85, 95%CI = 1.75–4.63, P < 0.001).

**Table 2:** Multiple binary logistic regression on factors significantly associated with suicide attempt among Chinese individuals receiving methadone maintenance treatment for heroin dependence.

Variables		One-month suicide attempt					Lifetime suicide attempt				
		Coefficient	Standard error	Wald χ2	P	OR(95%CI)	Coefficient	Standard error	Wald χ2	P	OR(95%CI)
Gender	Male	1					1				
	Female	1.216	0.500	5.904	0.015	3.37(1.27,8.99)	1.034	0.261	15.707	<0.001	2.81(1.69,4.69)
Age (years)	40–59	1					1				
	20–39	1.603	0.477	11.315	0.001	4.98(1.95,12.66)	1.006	0.253	15.781	<0.001	2.73(1.66,4.48)
Education	Middle school or higher	1					1				
	Primary school or lower	0.993	0.540	3.376	0.066	2.70(0.94,7.81)	0.729	0.369	3.909	0.048	2.07(1.01,4.27)
Marital status	Married	1					1				
	Non-married	1.583	0.472	11.288	0.001	4.88(1.93,12.35)	0.062	0.245	0.063	0.801	1.06(0.66,1.72)
Employment	Yes	1					1				
	No	0.612	0.464	1.740	0.187	1.84(0.74,4.58)	0.101	0.237	0.180	0.671	1.12(0.70,1.76)
Self-rated economic status	Good	1					1				
	Fair	0.020	0.586	0.001	0.973	1.02(0.32,3.21)	−0.219	0.290	0.570	0.450	0.80(0.46,1.42)
	Poor	0.744	0.687	1.175	0.278	2.11(0.55,8.09)	1.117	0.373	8.970	0.003	3.06(1.47,6.35)
Route of past heroin administration	Smoking	1					1				
	Injecting	1.527	0.725	4.436	0.036	4.60(1.45,28.51)	1.070	0.381	7.887	0.005	2.92(1.38,6.13)
Duration of heroin use (years)	≤10	1					1				
	>10	1.174	0.509	5.323	0.021	3.24(1.19,8.77)	0.228	0.238	0.924	0.337	1.26(0.79,2.00)
Duration of MMT (months)	≤24	1					1				
	>24	−0.563	0.430	1.711	0.191	0.57(0.25,1.32)	0.490	0.262	3.498	0.062	1.55(0.94,2.54)
Methadone dose (md/d)	≤70	1					1				
	>70	0.412	0.443	0.863	0.353	1.51(0.63,3.60)	0.318	0.234	1.851	0.174	1.37(0.87,2.17)
Depressive symptoms	No	1					1				
	Yes	0.625	0.574	1.184	0.277	0.54(0.17,1.65)	1.241	0.363	11.709	0.001	3.46(1.70,7.04)
Anxiety symptom	No	1					1				
	Yes	1.206	0.559	4.657	0.031	3.34(1.12,9.98)	0.632	0.295	4.602	0.032	1.88(1.06,3.35)
Pain	No	1					1				
	Yes	0.554	0.567	0.952	0.329	1.74(0.57,5.29)	0.383	0.272	1.991	0.158	1.47(0.86,2.50)
Loneliness	No	1					1				
	Yes	1.612	0.525	9.439	0.002	5.02(1.79,14.03)	0.252	0.245	1.055	0.304	1.29(0.80,2.08)
Antisocial personality disorder	No	1					1				
	Yes	1.768	0.502	12.422	<0.001	5.86(2.19,15.66)	1.047	0.248	17.739	<0.001	2.85(1.75,4.63)

## Discussion

To the best of our knowledge, this is the first large-scale study in China examining the prevalence and correlates of suicide attempt in IHD receiving MMT. We found 9.5% and 34.2% of the methadone-maintained IHD attempted suicide during the past month and their lifetime, respectively. The two prevalence figures indicate an extremely high prevalence of suicide attempt in Chinese IHD, because the lifetime prevalence, even the one-month prevalence of suicide attempt in IHD is much higher than the lifetime prevalence in the Chinese general population, which is estimated to be 0.8% only^49^. Compared to existing Chinese studies, the lifetime prevalence in our study is higher than that in the five studies with samples of IHD in mainland China (0.66–30.7%)^33–37^ and one study with a sample of IHD in Taiwan (17.8%)^50^. Nevertheless, our lifetime prevalence estimate still falls within the upper end of the lifetime prevalence range reported by Western studies with samples of patients with opioid dependence^10,20–25^. In addition, the one-month prevalence of attempted suicide in our sample is similar to that of IHD in Taiwan (10.9%)^50^, but higher than that of IUH in Australia (5.0%)^10^. These discrepancies could be attributed to differences in the assessment of suicide attempt (i.e., self-report vs. interview), treatment modality (i.e., MMT vs. inpatient), clinical settings (i.e., voluntary vs. compulsory detoxification institutions), type of opioid used (i.e., opioids vs. heroin), and patient characteristics (i.e., acute vs. prolonged withdrawal phase). Our data suggest that Chinese methadone-maintained IHD are at very high risk for suicide attempt.

In our sample, 51.1% were not married, 47.4% were unemployed, 23.2% had poor financial status, 33.8% were depressed, 36.3% were anxious, 53.6% suffered from pain, and 55.9% felt lonely. Therefore, a considerable portion of the IHD are socially and economically disadvantaged and have psychological and physical health problems. In addition, owing to the lack of mental health professionals and social workers in MMT clinics in China, psychosocial problems often go under-recognized and under-treated^11,51^, which further exacerbates the mental health of IHD. Because of these prevailing risk factors for suicidal behaviors, a very high prevalence of suicide attempt in IHD of MMT clinics is not surprising.

Overall, our findings on demographic and psychosocial correlates of suicide attempt in IHD are similar to those of the general population^48,52–55^. Because women have lower suicidal intent and use less lethal suicide methods than men, attempted suicides occur primarily among women^56,57^. This explains the higher prevalence of suicide attempt in female than in male IHD in our study. In general, compared to old age groups, attempted suicide in adolescents and young adults is characterized by greater attempt rate and higher attempt/completion ratio^58,59^. Accordingly, young IHD were more likely to survive due to attempted suicide but old IHD were more likely to die due to completed suicide. In this case, the old IHD may represent a special subgroup of IHD, who were less susceptible to suicide. In keeping with this possible survival bias, we found a higher rate of attempt in young than old age groups (20–39 vs. 40–59 years) in IHD. Because the two age-groups were born in different years, the age-group difference in rates of suicide attempt may also suggest the existence of a birth-cohort effect on the risk of attempted suicide among IHD. In addition, this study measured suicide attempt by a self-report question, so our measure of suicide attempt might be subject to recall bias. Due to age-related memory decline, the recall bias is likely to be more substantial in old than young age groups. The possible birth-cohort effect and recall bias may also explain the observed lower prevalence of suicide attempt in old than young IHD in this study. Because individuals with marital status of “other than married” may have inadequate spousal support to buffer against the negative effects of stressful life events^60^, non-married individuals receiving MMT may be more likely to attempt suicide when they have difficulties in daily life.

In this sample of IHD under MMT, we replicated the association between attempted suicide and a low socioeconomic status in the general population, as indicated by low levels of education and income^61^. Qualitative studies have shown that socioeconomically disadvantaged persons are more likely to experience negative life events across their life course, have negative emotions such as anger and shame, and hold a pessimistic attitude about future^62^. As a result of these, the significant relationships of suicide attempt with a low level of education and poor economic status in IHD is expected.

In line with prior studies with samples of patients with opioid dependence and community-adults^21,22,50,63–65^, we ascertained the elevated risk of attempted suicide in IHD with loneliness, depression and anxiety. Borderline personality disorder (BPD) is a commonly reported personality risk factor for attempted suicide in the literature^63,66^. However, because suicidal behaviors and self-harm are one of the diagnostic criteria for BPD, the cross-sectional BPD-attempt association is very likely to be spurious, as a result of the overlapping constructs between BPD and suicide attempt. Therefore, BPD was not assessed in the present study. We found that APD was significantly associated with an increased risk of suicide attempt in IUH, which is rarely reported in the literature. This association may be ascribed to the antisocial traits of IHD with APD such as impulsivity and aggression^67^, because a higher degree of aggression/impulsivity is a risk factor of attempted suicide in both opioid users and general population^24,68^.

Neither MMT duration nor dose of methadone was kept in the final multiple logistic regression model, despite their significant associations with lifetime suicide attempt in the univariate analyses. This finding may suggest that only providing MMT to IHD in MMT clinics may not be effective to reduce attempted suicides, because its effect can be easily masked or offset by other risk factors such as depression and anxiety. We found that patients who previously injected heroin and had a long duration of heroin use were at higher risk for attempted suicide. Evidence has shown that injecting drug users have very high prevalence of HIV, HCV, and HIV-HCV co-infection, and both HCV and HIV/AIDS has been linked to suicidal behaviors^69–71^. Duration of past heroin use can be viewed as a cumulative measure of past exposure to heroin, and long-term heroin use has been associated with more physical and mental health problems^72^. We speculate that the significant links between suicide attempt and injection heroin use and a long duration of heroin use might be due to these infectious diseases and deleterious health of injection users and long-term users.

This study has some limitations. First, detailed data on suicide attempt such as attempt methods, level of intention to die, total number of previous attempts, and age at first attempt were also important for suicide prevention, but we did not collect such data. Second, our sample of IHD was recruited from MMT clinics only, IHD of other settings, i.e., communities, compulsory detoxification centers, and hospitals, were not included, limiting the generalizability of our study findings. Third, due to the observational nature of this cross-sectional study, the causal relationships between suicide attempt and correlates could not be ascertained. Fourth, this study used self-rating scales to assess severities of depressive and anxiety symptoms. No clinical diagnostic interviews were conducted to ascertain the etiology of these symptoms, i.e., primary, secondary to physical illnesses, or induced by heroin dependence. This information is also important for the clinical implication of the study findings. Fifth, as we mentioned above, this study may be subject to recall bias due to the use of a self-report measure of suicide attempt. We may underestimate the prevalence of suicide attempt in this sample of IHD. Sixth, due to limitations in the study design, a secondary data analysis, some potentially important risk factors of attempted suicide such as medical illnesses, alcohol dependence, and psychotic symptoms, were not included in our analysis. It should be noted that, because China’s clinical guideline for MMT does not recommend MMT for IHD with alcohol dependence^73^, no patients with alcohol dependence were identified in the three MMT clinics of our study. Recruiting patients from other clinical settings such as compulsory detoxification institutions and community-based rehabilitation centers for IHD would solve this issue. Finally, we ran the multivariable logistic regression twice to identify factors associated with suicide attempt but did not correct for multiple testing because the analysis was exploratory in nature. Findings of some correlates might be false positive. Longitudinal studies that assess detailed characteristics of suicide attempt, include more candidate risk factors such as alcohol dependence, and recruit samples of IHD from diverse clinical settings are warranted to address these issues.

In conclusion, suicide attempt is highly prevalent among Chinese IHD of MMT clinics, indicating the high risk of suicide in this patient population. There is an urgent need for the mental health professionals to identify and address the epidemic of suicide attempt and other suicidal behaviors in MMT clinics in China. Among IHD, suicide attempt is associated with a range of demographic, psychosocial, and clinical factors. Given the strong predictive value of attempted on completed suicide, efforts to prevent or reduce suicides in MMT clinics may be effective to target on those who are females, are young, are not married, have a low socio-economic status (i.e., a low level of education, poor financial status), inject heroin before MMT, have a long duration of heroin use, feel lonely, have clinically significant depressive and anxiety symptoms, and have APD. Services for IHD in MMT clinics should include regular screenings for those at risk for attempted suicide and other suicidal behaviors, expanded psychosocial supports, and, when necessary, psychiatric assessment and treatment and crisis intervention.

## Supplementary information

Supplementary Table 1: Checklist for assessing the eligibility of patients

## Data Availability

The datasets used and/or analyzed during the current study are available from the corresponding author on reasonable request.
