# Supplementary material for: Prevalence and correlates of suicide attempt among Chinese individuals receiving methadone maintenance treatment for heroin dependence
Source: Sci Rep. 2019 Oct 30;9:15859. doi: 10.1038/s41598-019-52440-x (PMC6821703; doi:10.1038/s41598-019-52440-x)
Supplement: Supplementary file 1 — Supplementary Table 1: Checklist for assessing the eligibility of patients [file 41598_2019_52440_MOESM1_ESM.docx]

**Prevalence and correlates of suicide attempt among Chinese individuals receiving methadone maintenance treatment for heroin dependence**

Bao-Liang Zhong^1,2,3^, Wu-Xiang Xie^4^, Jun-Hong Zhu^3^, Jin Lu^5^, and Hongxian Chen^1,2*^

^1^Department of Psychiatry, The Second Xiangya Hospital, Central South University, Changsha, Hunan, China

^2^China National Clinical Research Center on Mental Disorders (Xiangya), Changsha, Hunan, China

^3^Affiliated Wuhan Mental Health Center, Tongji Medical College of Huazhong University of Science & Technology, Wuhan, Hubei Province, China

^4^Peking University Clinical Research Institute, Peking University Health Science Center, Beijing, China

^5^Department of Psychiatry, The First Affiliated Hospital of Kunming Medical University, Kunming, Yunnan Province, China

*Correspondence to: Hongxian Chen, Department of Psychiatry, The Second Xiangya Hospital, Central South University, No. 139, Middle Renmin Road, Changsha 410011, Hunan, China. Tel.: +8613875970393. Email: shenhx2018@csu.edu.cn

**Supplementary Material**

**Supplementary Table 1: Checklist for assessing the eligibility of patients**

| **A** | **B** | **C** |
| --- | --- | --- |
| **Inclusion criteria** |  |  |
| 1. Age (years):≥20 | Yes □ | No □ |
| 2. Lifetime DSM-IV diagnosis of heroin dependence | Yes □ | No □ |
| 3. Taking liquid methadone | Yes □ | No □ |
| 4. Voluntary to participate and signed the informed consent form | Yes □ | No □ |
| **Exclusion criteria** |  |  |
| 5. Current DSM-IV diagnosis of alcohol dependence | No □ | Yes □ |
| 6. Diagnosis of brain organic mental disorders (__________________) | No □ | Yes □ |
| 7. Having any psychotic symptoms: hallucination □ delusion □ others (________) | No □ | Yes □ |
| 8. Is the patient too physically ill to participate in the study?  Please record specific physical conditions: a.______, b.______, c.______, d.______, e.______ | No □ | Yes □ |

**Please count ticks in boxes of the column C:**

**0 □ eligible; ≥1 □ ineligible**
